# Supplementary material for: Triplet correlations among similarly tuned cells impact population coding
Source: Front Comput Neurosci. 2015 May 18;9:57. doi: 10.3389/fncom.2015.00057 (PMC4435073; doi:10.3389/fncom.2015.00057)
Supplement: Supplementary file 1 [file Presentation1.PDF]

Supplementary Material: Triplet correlations among similarly tuned cells impact population coding

Alex Cayco-Gajic [1,*], **Joel Zylberberg** [1] **and Eric Shea-Brown** [1]

[1] *Department of Applied Mathematics, University of Washington, Seattle, WA, USA*

Correspondence*:
Corresponding Author
Department of Applied Mathematics, University of Washington, Lewis Hall #202
Box 353925, Seattle, WA, 98194, USA, aligator@uw.edu

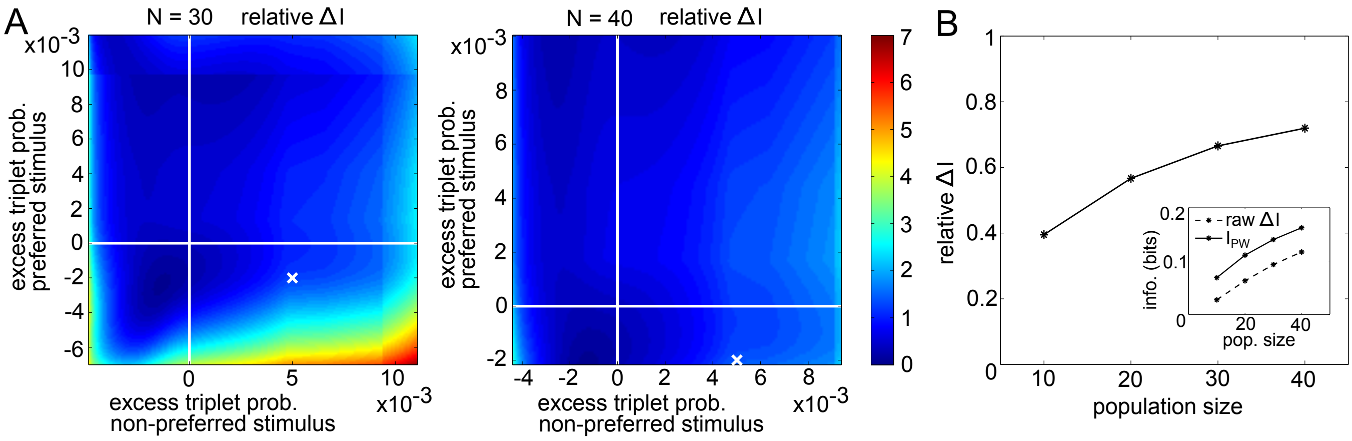

Supplementary Figure 1. Supplementary figure S1. Effect of population size on mutual information in homogenous populations. (A) Relative increase in information as the excess triplet probability is varied for responses to the preferred and non-preferred stimuli, shown for populations of $N = 30$ (left) and $N = 40$ (right) cells. Note that the range of triplet correlations is smaller for the larger population. This is because, as N increases, there are tighter constraints on the possible values of triplet correlations that can be attained homogeneously across every triplet in the population, while still maintaining the same (low) predefined firing rates and pairwise correlations.Still, in the region of overlap, the strength of the impact on mutual information is similar in magnitude in both plots. Here, firing rates and pairwise correlations are fixed to: $\mu_1 = 0.25$, $\mu_2 = 0.35$, $\rho = 0.05$. Compare with the plot of raw mutual information (as opposed to the relative increase in information) in 10-cell populations that is shown in Figure 3A. (B) Relative increase in information (black curve) for fixed triplet correlations and lower-order statistics, for increasing population size. Specifically, the values of the triplet correlations were: $\kappa = .005$ for the non-preferred stimulus, and $\kappa = -.002$ for the preferred stimulus, corresponding to the cross in panel A. The impact of the triplet statistics on mutual information grows with population size. Inset shows I_{PW}, the mutual information between the pairwise distribution and the stimuli (solid line) and ΔI, the raw increase in information due to triplet correlations (dashed line) for varying population size.
